# Does standard phisiotherapy influence the health-related quality of life of JIA patients?

**DOI:** 10.1186/1546-0096-9-S1-P208

**Published:** 2011-09-14

**Authors:** TMS Mendonça, CA Len, MTRA Terreri, CHM Silva, RMC Pinto

**Affiliations:** 1 Department of Pediatry, Federal Univerisity of São Paulo – Paulista School of Medicine, Brazil; 2 Department of Pediatry, Federal Univerisity of Uberlandia, Brazil

## Background

The assessment of health-related quality of life (HRQL) in the perception of patients with chronic diseases such as juvenile idiopathic arthritis (JIA), reflects the effectiveness of the interventions to which those patients have been submitted.

## Aim

To assess the HRQL of the JIA patients participating in a standard physiotherapy program, according to the arthritis subtype.

## Methods

Clinical trial of a consecutive sample of 26 JIA (ILAR) patients of the Oligoarticular (46,2%), Polyarticular (23,1%) and Systemic (30,7)% subtypes, with an average 11,2±4,1 years of age. The patients participated in a standard physiotherapy program twice a week for 6 months. The Chi-square test has been used in order to evaluate the attendance to the program. The HRQL has been assessed by means of the PedsQL 4.0 and the Kruskal-Wallis test with *post hoc* the Duunn test has been used in order to compare the median and percentiles (25 and 75) of the scores of these instruments in the baseline and end-of-6-months periods.

## Results

The attendance of the patients to the submitted program has been significant (p<0,05) with an average of 45±1,1 of the 48 sessions proposed to each patient. A significant difference has been detected (p<0,05) between the median and percentiles (25 and 75) of the oligoarticular subtype in the in the psychosocial summary of 46,7 (39,2; 68,3) versus 70,7(55;72) and in the overall score of the PedsQL 4.0 of 49,1 (39,4; 65,8) versus 72,2 (50;75) in the baseline and end-of-6-months periods.

## Conclusion

The standard physiotherapy program has promoted a positive impact in the HRQL of JIA patients of the Oligoarticlar subtype. The psychosocial aspects have been responsible for this impact. Randomized, controlled studies with a larger patient sample are necessary for the inference of these results. The perception of the patients in relation to the therapeutic measures they are submitted to is essential for the success of the treatment.

